# Tapinarof for the treatment of psoriasis

**DOI:** 10.1111/dth.15931

**Published:** 2022-10-21

**Authors:** Sofia Nogueira, Maria Alexandra Rodrigues, Ron Vender, Tiago Torres

**Affiliations:** ^1^ Instituto de Ciências Biomédicas Abel Salazar University of Porto Porto Portugal; ^2^ Department of Dermatology Centro Hospitalar do Porto Porto Portugal; ^3^ Dermatrials Research Inc Hamilton Canada; ^4^ McMaster University Hamilton Canada

**Keywords:** aryl hydrocarbon receptor, GSK2894512, psoriasis, Tapinarof, topical treatment

## Abstract

Although topical drugs are the mainstay of treatment for patients with mild‐to‐moderate psoriasis, the developments observed in this field in the last two decades have been limited. The most commonly used drugs are still vitamin D analogues and corticosteroids, both with several limitations. The aryl hydrocarbon receptor (AhR) plays a role in the pathogenesis of psoriasis, and tapinarof, a novel, first‐in‐class, small molecule topical therapeutic AhR‐modulating agent has been recently approved by the FDA for the topical treatment of plaque psoriasis in adults. Two large, 12‐week, phase III trials, PSOARING 1 and 2, showed that 35.4%–40.2% of patients in the tapinarof 1% cream arm achieved the primary endpoint (Physician's Global Assessment [PGA] score of 0 or 1 and a decrease of ≥2–5 points at week 12) compared with 6.0%–6.3% for vehicle arm, respectively. The most common adverse effects were folliculitis, contact dermatitis, headache and pruritus. In the open label, 40‐week, extension trial, PSOARING 3, the efficacy and safety results were similar, with 40.9% of patients achieving a PGA = 0 at least one time during the trial and 58.2% of patients with PGA≥2 achieved PGA = 0/1 at least once during the trial, without tachyphylaxis. There were no new safety signals, with most frequent adverse events being folliculitis, contact dermatitis, and upper respiratory tract infection. Tapinarof 1% cream has shown to be effective and to have a favorable safety profile in the treatment of psoriatic patients, representing an alternative to the current therapeutic options, increasing our armamentarium in the topical treatment of psoriasis.

## 1 INTRODUCTION

Psoriasis is a chronic inflammatory skin disease with an estimated incidence of 1%–3% worldwide.^1^ Plaque psoriasis is the most common form and is characterized by erythematous scaly plaques in specific sites, with periods of remission and exacerbation.^2^ Its pathophysiology is multifactorial; the IL‐23/IL‐17A pathway is considered the central pathogenic axis and has been the target for many highly effective biologic drugs, which has been a major progress in the treatment of psoriasis patients.^2^ However, most of patients suffer from mild, localized disease, having no requirement for systemic therapy as a first line treatment. Therefore, topical treatment is essential in the management of these patients.^2^ Currently available topical therapies include corticosteroids, vitamin D analogues (calcipotriene or calcitriol), tazarotene and coal tar. However, these medications have several adverse effects that limit their use in psoriatic patients, in both the short and long‐term.^3^ Unfortunately, advances in the recent years have been limited, and essentially related to new formulations, with no new mechanisms of action to be approved, meaning the adequate topical treatment of psoriasis remains an important unmet need.^4^

Recently, a new topical therapy called tapinarof (GSK2894512) was developed and is currently approved by the FDA for the topical treatment of plaque psoriasis in adults.^5^ Tapinarof is a novel, first‐in‐class, small molecule topical therapeutic aryl hydrocarbon receptor (AhR)‐modulating agent that binds and activates AhR, reduce skin inflammation, normalize the skin barrier, reduce oxidative stress and regulate gene expression in immune cells.^6^

This article reviews the role of AhR on psoriasis and summarizes the data on the efficacy and safety of tapinarof cream 1% in the treatment of psoriasis. A search in the PubMed database (up until June 2022) for articles with the specific keywords: “psoriasis,” “tapinarof,” “GSK2894512,” “aryl hydrocarbon receptor,” and “topical treatment” were carried out. The articles were selected by the relevance of the abstract and established objectives. When pertinent, the bibliographic references present in the selected articles were also included.

## 2 THE ROLE OF ARYL HYDROCARBON RECEPTOR ON PSORIASIS

Aryl hydrocarbon receptor (AhR) is a cytosolic ligand‐dependent transcription factor expressed in different types of skin cells (keratinocytes, fibroblasts, mastocytes, and melanocytes).^6^, ^7^, ^8^, ^9^ In healthy skin, AhR signaling is essential for maintaining skin homeostasis through regulating the skin immunological network, keratinocyte differentiation, skin barrier function, and gene expression in cells.^10^, ^11^ This receptor can bind to endogenous or exogenous agents such as pollutants, polycyclic aromatic hydrocarbons (PAHs), microbial products, dietary ligands, and phytochemicals that activate the AhR.^8^, ^12^, ^13^, ^14^ When a ligand binds to the receptor, the AhR‐ligand complex migrates to the nucleus and dimerizes by the AhR nuclear translocator (ARNT).^9^, ^12^ The AhR‐ligand‐ARNT complex attaches to specific DNA recognition sites, causing gene transcription.^9^, ^12^

AhR plays a role in the pathogenesis of inflammatory skin diseases.^11^, ^15^ Two AhR signaling pathways have been described: the canonical activation pathway (AHR: ARNT signaling), which induces carcinogenesis, hyperpigmentation and disables apoptosis and the non‐canonical AhR signaling pathway (includes other pathways such as EGFR, MAPK, NF‐κB, STATs, and NRF‐2), which induces inflammation, abnormal barrier function, hypopigmentation, production of cytokines and oxygen reactive species.^16^ This pathway has been recognized as a potential therapeutic target.^11^, ^16^

In psoriatic skin, the aryl hydrocarbon receptor is present in cutaneous plaques and regulates the inflammatory response as well as the terminal differentiation of keratinocytes and T lymphocytes.^11^, ^12^ It was shown in vitro and in vivo studies AhR deficit causes increased responsiveness to pro‐inflammatory stimuli and the development of uncontrolled inflammation. AhR‐deficient mice experienced marked psoriasiform skin inflammation with elevated IL‐17 and IL‐22 production in an imiquimod‐induced psoriasis model compared to controls. In addition, also dysregulation of keratinocytes and fibroblasts was observed in the deficient mouse skin models. However, IL‐17 is not the only cytokine involved in the development of psoriasiform inflammation in the AhR‐deficient mice model, as inflammation was not was not attenuated when IL‐17 was blocked.^14^

**FIGURE 1:**
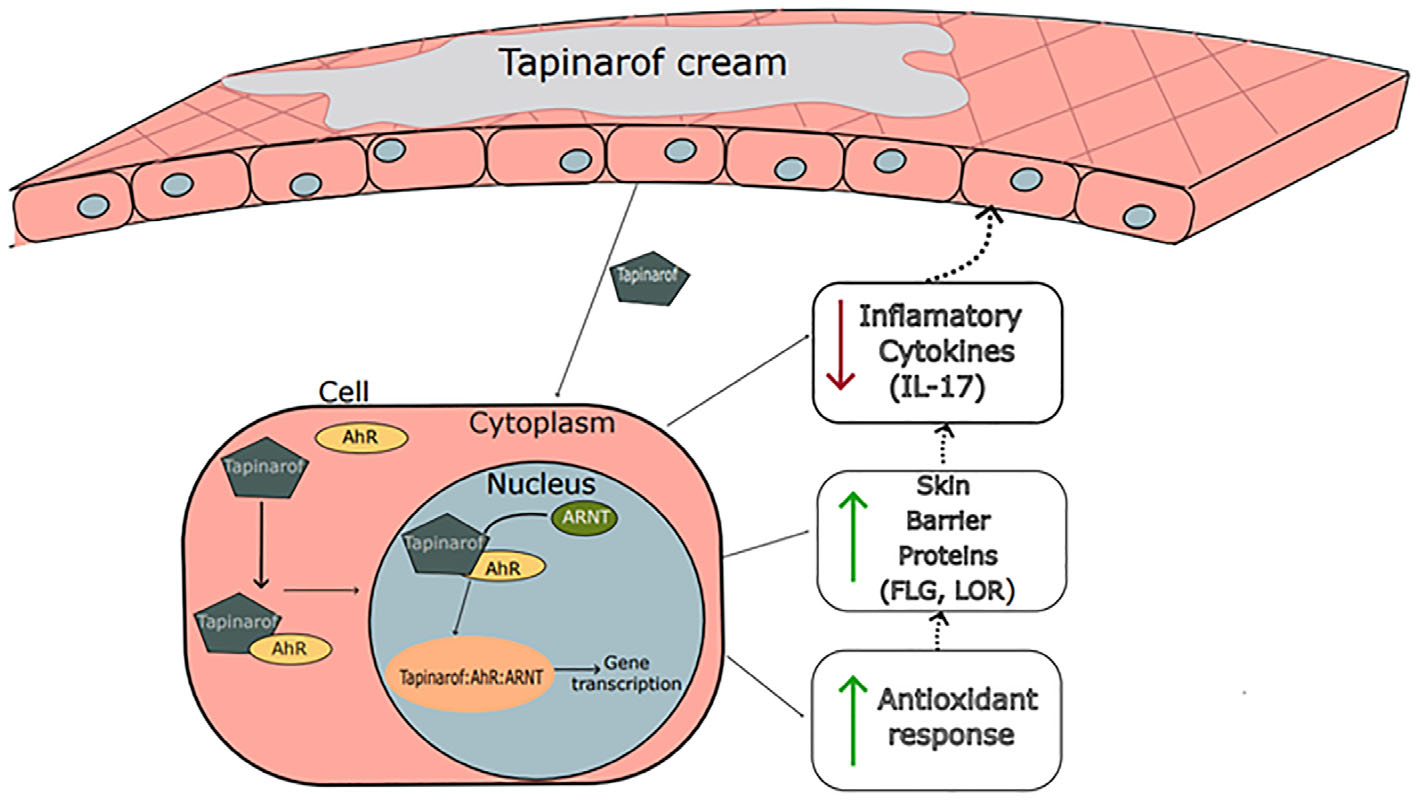
Tapinarof mechanism of action

Tapinarof activates the nuclear factor erythroid 2‐related factor 2 (NRF‐2) pathway and consequently produces antioxidative enzymes that limit oxidative stress (Figure 1).^6^, ^10^, ^12^ It induces the production of loricrin (LOR) and filaggrin (FLG), by activating transcription factor OVO‐like 1 (OVOL1); these proteins play an important role in maintaining skin barrier function (Figure 1).^6^, ^10^ AhR activation by tapinarof regulates the differentiation of T‐helper (Th17) and T regulatory cells, leading to the downregulation of IL‐17 (Figure 1) and consequently reducing inflammation in psoriatic skin.^10^ The mechanism of action of tapinarof also depend on its effect on the microbiome of the skin, as tapinarof as shown some antimicrobial activity with consequente attenuation of cytokine production and improvement of the disease.^17^

In conclusion, there is growing evidence that AhR plays a vital role, beyond the one present in skin homeostasis and integrity, as a potential therapeutic target in inflammatory diseases.^16^ The AhR ligand and the cell environment are crucial elements that can influence AhR‐driven signals differentially, functioning as inhibitors or stimulators of inflammatory processes.^8^

## 3 TAPINAROF

Tapinarof is a topical aryl hydrocarbon receptor modulator of bacterial origin.^12^ Its effects were observed from the interactions of the nematode of the genus *Heterorhabditis* with insects. This nematode carries in its gut gram‐negative bacteria with luminescence properties called *Photorhabdus luminescens*, that is, released when the nematode infects an insect, killing it, by producing metabolites.^12^, ^18^, ^19^ It was observed that insects infected by this nematode did not putrefy after death, in contrast to the fast degradation observed in the absence of the parasite.^12^, ^18^, ^20^ In fact, *P. luminescens* produces metabolites with antibacterial properties that responsible for the observed biological action.^12^, ^19^ One such metabolite, 3,5‐dihydroxy‐4‐isopropylstilbene (tapinarof), was isolated and identified.^12^, ^21^ Tapinarof binds directly to AhR, resulting in the suppression of inflammatory cytokines, the modulation of skin barrier protein expression, and antioxidant activity.^12^, ^22^

Tapinarof cream 1% (GSK‐2894512) has the same active principle as benvitimod cream 1% (WBI‐1001). Benvitimod was developed in China, via a separate clinical trials program. It requires a twice daily application has a different excipients/formulation and is approved for topical use in China since 2019 for patients with mild‐to‐moderate plaque psoriasis.^23^, ^24^, ^25^, ^26^ Our review only focuses on tapinarof cream 1% (GSK‐2894512).

### 3.1 Pharmacodynamics and pharmacokinetics

A phase I study evaluated the pharmacokinetics (PK) of tapinarof cream 1% and 2% applied twice daily in 11 participants with atopic dermatitis. It was shown that a higher concentration of tapinarof cream leads to greater plasma concentrations of the drug, which tend to decrease over time which proves there is no accumulation after the repeated exposure. On day 1, the mean maximum serum concentration ($C_{\max})$ for tapinarof 2% and 1% was 2.5 and 1.2 ng/ml, respectively. At day 21 it was 0.3 ng/ml for the 2% cohort and 0.15 ng/ml for the 1% cohort. The value of the median time to reach the maximum serum concentration ($T_{\max})$ for both concentrations of tapinarof cream were between 1 and 4 h.^22^

Regarding the pharmacokinetic profile of tapinarof 1% applied once daily, it was observed that the mean concentration was 898 pg/ml on day 1, decreasing over time measuring a mean concentration of 116 pg/ml on day 29, which might be due to the skin's barrier function being restored. The plasma concentration of tapinarof 1% cream hits $T_{\max}$ in a mean of 4, 49 h, within a range of 2–5 h post administration of the first dose.^27^

Additionally, biodistribution and residency of tapinarof 1% cream were evaluated in a phase I trial that included six healthy volunteers and used fluorescence lifetime imaging microscopy (FLIM), a non‐invasive method limiting the use of skin biopsies. The cream was applied for 7 days, and images were taken every day before the drug administration. It was shown that in the first few days, the cream remained in the superficial layers of the skin, at day 10, there was no fluorescence detected.^28^

Tapinarof metabolism is hepatic, with cytochrome (CYP) P450 playing a predominant role. The primary enzymes involved are CYP1A2 and CYP3A4. Conversely, CYP2C9, CYP2C19, CYP2C20, CYP2D6 play minimal roles in the oxidative metabolism of tapinarof in hepatocytes.^22^ Due to the large number of CYP enzymes involved in tapinarof metabolization, inhibiting one CYP enzyme would unlikely result in increased tapinarof exposure.^22^

### 3.2 Efficacy

A phase IIa, multicenter, open‐label trial (NCT04042103) (Table 1) that assessed the efficacy of tapinarof in treating patients with extensive plaque psoriasis included 21 adult patients. Tapinarof cream 1% was applied once daily for 29 days to all the affected areas. The efficacy was evaluated by comparing the patient global assessment (PGA), Psoriasis Area and Severity Index (PASI) and the percentage of body surface area (BSA) on day 1 and day 29. At baseline, 12 patients had a PGA of 3 and 7 patients a PGA of 4. The mean of PASI was 24.65 (SD: 7146). On day 29, 14 patients (73.7%) had *a* ≥ 1 grade PGA improvement, and six patients had a ≥ 2 grade improvement compared to day 1. Four patients (21.1%) achieved a PGA score of 0 or 1 and ≥2 grade improvement in PGA. Also, on day 29, there was a significant mean change in PASI score to 15.14 (95% CI – 19.61 to – 10.66). Eighteen patients improved their PASI score during this trial. Seven patients achieved PASI75. On day 1, the mean percentage BSA was 27.20 (SD: 7481). On day 29, there was a change in the mean percentage BSA to 14.44 (95% CI – 19.84 to – 9.04). Nineteen completed the trial. The reasons for discontinuation were “lost to follow‐up” in one patient and withdrawal from the study in another.^27^

**TABLE 1:** Clinical trials on efficacy and safety of tapinarof

Study	Drug	Design	Outcomes	Results	Safety
NCT04042103^27^ (Phase IIa, multicenter, open‐label trial)	Tapinarof 1% cream QD	‐ 21 participants (adults) with: ‐plaque psoriasis ‐PGA ≥3 ‐BSA ≥20% ‐ 19 participants completed the trial ‐ Tapinarof cream 1% applied QD for 29 days. There was no restriction on the area of application	*Primary*: Evaluate the safety, tolerability, efficacy (PGA, PASI and BSA) and PK of tapinarof cream 1% QD *Secondary*: Exclude clinically relevant effects of tapinarof cream 1% QD on the QT interval	*Improvement in PGA score on day 29*: ≥ 1‐grade: 14 patients (73.7%) ≥ 2‐grade: 6 patients (31.6%) PGA 0 or 1 and ≥2‐grade: 4 patients (21.1%) *PASI*: Mean day 1: 24,65 Mean day 29: 15,14 PASI75: 7 patients (36.8%) *% BSA*: Mean day 1: 27,20 Mean day 29: 14,44	*Treatment‐emergent adverse effects* Folliculitis (19%) Headache (19.0%) Others: Back pain Pruritus Contact dermatitis
Robbins et al^29^ (Phase II, randomized, double‐blind, vehicle‐controlled study‐NCT02564042)	Tapinarof 0.5% or 1% cream QD or BID	227 patients aged 18–65 with: ‐Stable plaque psoriasis (≥6 months) ‐BSA ≥1%–≤15% ‐PGA score ≥2 Randomized in 6 arms (1:1:1:1:1:1, 12 weeks): 1% tapinarof BID (*n* = 38) 1% tapinarof QD (*n* = 38) 0.5% tapinarof b.i.d (*n* = 38) 0.5% tapinarof QD (*n* = 38) Vehicle b.i.d (*n* = 38) Vehicle QD (*n* = 38) 175 patients completed the trial	*Primary* % of patients with a PGA score of 0 or 1 at week 12 ≥2‐grade improvement in PGA score from baseline *Secondary* % patients achieving PASI 75 Mean change in PASI and BSA Changes in PGA Evaluation of adverse effects	*PGA response at week 12*: Higher (*p* = 0.05) in tapinarof groups 65%–1% b.i.d 56%–1% QD 46%–0.5% b.i.d 36%–0.5% QD *PASI 75 at week 12*: Higher (*p* = 0.05) in tapinarof groups 65%–1% b.i.d 56%–1% QD 46%–0.5% b.i.d 46%–0.5% QD 16%—vehicle b.i.d 5%—vehicle QD *Mean reduction in %BSA at week 12*: Tapinarof group: 3.6%–4.9% Vehicle group: 1%–1.6%	*Treatment‐emergent adverse events*: 68%–1% b.i.d 53%–1% QD 58%–0.5% b.i.d 45%–0.5% QD 24%—vehicle b.i.d 26% vehicle QD Mild‐to‐moderate intensity *Most common*: Folliculitis, contact dermatitis, application site dermatitis, application site, irritation, allergic dermatitis, headache, monocyte count decrease (1 case in vehicle group) *Discontinuation*: 10% tapinarof groups 1% vehicle groups. Due to contact dermatitis and application site dermatitis
Stein Gold L et al^30^ (Multicenter, phase IIb, double blinded, randomized, vehicle‐controlled study)		227 patients aged 18–65 with: ‐plaque psoriasis ‐BSA ≥1% ‐ ≤15% ‐PGA ≥2 Randomized 1:1:1:1:1:1 received for 12 weeks: ‐Tapinarof cream 0.5% or 1% QD or BID ‐vehicle cream QD or BID	*Efficacy outcomes*: PGA response Change in mean PGA Total target lesion grading scores % patients achieving PASI50, PASI75, and PASI90.	*PGA response*: Higher in all tapinarof cream groups compared with vehicle group *Mean improvements in PGA scores* from baseline: Higher in all tapinarof groups compared to vehicle. *Total target lesion grading* Higher reductions in total target lesion grading scores from baseline were observed in all tapinarof groups beginning at week 2. *PASI50, PASI75, and PASI90* Higher responses starting at week 2 compared to vehicle cream, and superior efficacy maintained until week 12 and 4 weeks after the treatment	*Treatment‐emergent adverse effects* Mild‐to‐moderate in severity *Tolerability scores*: *Patient*: Score of 0 or 1—none or slight application site burning/stinging and itching during 12 weeks *Investigator*: Predominantly 0 (no irritation) through 12 weeks No skin atrophy
	Tapinarof 1% cream 0.5% or 1% cream QD or BID	4 weeks of follow up			
PSOARING 1^31^ NCT03956355 (Phase III, multicentre, randomized, double blind, vehicle‐controlled trial)	Tapinarof 1% cream QD	−510 patients aged 18–75 with: ‐ mild to severe plaque psoriasis ‐BSA ≥3%–≤20% ‐PGA ≥2 2 arms (2:1, 12 weeks): −1% Tapinarof cream QD (*n* = 340) ‐Vehicle cream QD (*n* = 170)	*Primary* PGA response: PGA score of 0 or 1 and a decrease of ≥2–5 points at week 12 *Secondary* Changes in the baseline of BSA, PGA score of 0 or 1, PASI 75 and PASI 90 at week 12.	*Primary*: 35.4%—Tapinarof arm (*p* < 0,001 vs vehicle) 6%—Vehicle arm *Secondary*: *PASI 75* 36.1%—Tapinarof arm (*p* < 0.001 vs. vehicle) 10.2%—Vehicle arm *PGA score of 0 or 1* 37.8%—Tapinarof arm 9.9%—Vehicle arm (*p* < 0,001 vs vehicle) *BSA changes* −3.5%—Tapinarof arm (*p* < 0,001 vs vehicle) −0.2%—Vehicle arm *PASI 90* 18,8%—Tapinarof arm (*p* < 0,001 vs vehicle) 1,6%—Vehicle arm	*Treatment‐emergent adverse effects* Tapinarof arm: 50.3% Vehicle arm: 22.4% Most common: folliculitis, contact dermatitis, headache, pruritus
PSOARING 2^31^ NCT03983980 (Phase III, multicentre, randomized, double blind, vehicle‐controlled trial)	Tapinarof 1% cream QD	515 patients aged 18–75 with: ‐Plaque psoriasis ‐ BSA ≥3%–≤20% ‐ PGA score ≥2 2 arms (2:1, 12 weeks): −1% Tapinarof cream QD (*n* = 343) ‐Vehicle cream QD (*n* = 172)	*Primary* PGA response: PGA score of 0 or 1 and a decrease of ≥2–5 points at week 12 *Secondary* Changes in the baseline of BSA, PGA score of 0 or 1, PASI 75 and PASI 90 at week 12.	*Primary*: 40.2%—Tapinarof arm (*p* < 0.001 vs. vehicle) 6.3%—Vehicle arm *Secondary*: *PASI 75* 47.6%—Tapinarof arm (*p* < 0,001 vs vehicle) 6.9%—Vehicle arm *PGA score of 0 or 1* 43,6%—Tapinarof arm (p < 0,001 vs vehicle) 8.1%—Vehicle arm *BSA changes* −4.2%—Tapinarof arm (*p* < 0,001 vs vehicle) −0.1%—Vehicle arm *PASI 90* 20.9%—Tapinarof arm (*p* < 0,001 vs. vehicle) 2.5%—Vehicle arm	*Treatment‐emergent adverse effects* Tapinarof arm: 54.5% Vehicle arm: 26.2% Most common: folliculitis, contact dermatitis, headache, pruritus
PSOARING 3^32^ (multicenter, open label, extension study)	Tapinarof 1% cream QD	763 patients that had completed 12 weeks' treatment with tapinarof or vehicle in PSOARING 1 or 2 18–75 years with chronic plaque psoriasis, stable for at least 6 months before randomization %BSA affected of ≥3% and ≤ 20% (excluding scalp, palms, soles, fingernails, and toenails), and (PGA score of 2 (mild), 3 (moderate), or 4 (severe) at screening)	*Safety* Incidence and frequency of AEs, patient‐ and investigator‐assessed local tolerability, vital signs, physical examinations, and laboratory tests *Efficacy* PGA = 0 at any time during the trial; the total duration of remittive effect; the median duration of remittive effect in patients entering with PGA = 0; and the proportion of patients entering the trial with PGA≥2 who achieved a response (PGA = 0 or 1) at any time during the trial	*Efficacy* 40.9% of patients achieved complete disease clearance (PGA = 0) and 58.2% entering with PGA≥2 achieved PGA = 0 or 1. Mean duration of remittive effect off‐therapy for patients achieving PGA = 0 was 130.1 days.	*Safety* No new safety signals were observed. Most frequent adverse events were folliculitis (22.7%), contact dermatitis (5.5%) and upper respiratory tract infection (4.7%). *Tolerability* >90% of patients showed no irritation at all visits Good tolerability on sensitive and intertriginous skin. Patient‐reported burning/stinging and itching was low by 86%–92% of patients over 40 weeks
		Patients entering with PGA = 0 discontinued treatment and were monitored for remittive effect (maintenance of a PGA score of 0 or 1 while off therapy) Patients entering with PGA≥1 were instructed to apply tapinarof 1% QD	*Tolerability* Local tolerability was evaluated using a patient reported 5‐point scale of 0 (none) to 4 (strong/severe) for burning/stinging and itching, and an investigator‐assessed 5‐point scale of 0 (no irritation) to 4 (very severe) for dryness, erythema, and peeling		

Abbreviations: AE, adverse event; BID, twice daily; BSA, body surface area; PASI, Psoriasis Area and Severity Index; PASI75 ≥ 75% improvement in Psoriasis Area and Severity Index; PASI90 ≥ 90% improvement in Psoriasis Area and Severity Index; PGA, Physician's Global Assessment; QD, once daily.

A phase II, double‐blind, randomized, vehicle‐controlled study (NCT02564042) (Table 1) included 227 participants aged 18 to 65, with stable plaque psoriasis for more than 6 months, with a BSA between 1% and 15% and a PGA score ≥2. Patients were randomly assigned in a 1:1:1:1:1:1 ratio, receiving once or twice daily vehicle cream or tapinarof cream 0.5% or 1% for 12 weeks and a 4 week follow up after the end of study treatment. The primary outcome was PGA response, which included the proportion of patients achieving at week 12, a PGA score of 0 or 1 and at least an improvement of 2 points comparing to baseline. At week 12, statistical significance was achieved with a PGA score of 0 or 1 and PASI 75 in the tapinarof groups when compared with the vehicle treatment groups. This response was seen as early as week 2 of treatment, and maintained during 4 weeks of follow‐up.^29^

Additional outcomes were evaluated in a phase IIb (Table 1) study, which consisted of patients achieving PASI 50, PASI 75 and PASI 90, mean changes in PGA score and total target lesion grading score. At week 12, the percentage of patients who achieved PASI 50 was 71%–92% in the tapinarof group compared to 10%–32% in the vehicle group (all *p* < 0.001). PASI 75 was reached at week 12 in 65% (*p* = 0.001) and 56% (*p* < 0.001) in the tapinarof 1% group, twice or once daily, respectively. In the 0,5% tapinarof group PASI 75 was achieved in 46% in both twice or once daily applications (*p* = 0.035, and *p* = 0.002, respectively). In the control group, 16% (twice daily) and 5% (once daily) reached PASI 75. For PASI 90 39% (*p* = 0.002) and 40% (*p* = 0.001) of participants in the 1% tapinarof group, and 31% (*P* = 0.008) and 18% (*P* = 0.057) of participants in the 0.5% tapinarof group, twice or once daily respectively. In the vehicle group, 0% of participants reached PASI 90 at week 12. In addition, changes in the mean PGA from baseline and the PGA response rate, was more significant in all tapinarof groups, and maintained for 4 weeks after the end of the study. Lastly, in evaluating the total target lesion grading scores from baseline, there was a higher reduction in the tapinarof groups than vehicle, beginning at week 2 and maintained until week 16.^30^

Two phase III multicentre, randomized, double blind, vehicle‐controlled trials, PSOARING 1 (NCT03956355) and PSOARING 2 (NCT03983980), included participants aged between 18 and 75 years old with mild‐to‐severe plaque psoriasis with stable disease for at least 6 months and BSA between 3% to 20% and a PGA score of at least of 2. PSOARING 1 (Table 1) study had 510 participants and PSOARING 2 (Table 1) had 515 participants. The subjects were randomized in a 2:1 ratio, receiving vehicle cream or 1% tapinarof cream once daily for 12 weeks. The primary outcome was PGA response (PGA score of 0 or 1 and at least 2‐grade improvement from baseline at week 12). In both studies, a significantly higher proportion of patients in the tapinarof arm met the primary endpoint than vehicle cream at week 12. In PSOARING 1, the proportion of subjects achieving PGA response was 35.4% in the tapinarof 1% cream arm (*p* < 0.001 vs. vehicle) and 6.0% for vehicle cream. In PSOARING 2, the percentage of subjects achieving the primary endpoint was 40.2% for tapinarof 1% cream (*p* < 0.001 vs vehicle) and 6.3% for vehicle cream. Overall, in the two studies, at week 12, PASI 75, a PGA score of 0 or 1, BSA changes and PASI 90 were observed in a significantly higher proportion of patients in the tapinarof 1% cream arm.^31^

Recently, the first long‐term results were published from the PSOARING 3 trial. This open label, extension trial with 763 patients aimed to explore the one‐year efficacy, safety, tolerability, durability of response on therapy (absence of tachyphylaxis), and duration of remittive effect off‐therapy of tapinarof cream 1% in plaque psoriasis. Patients received a maximum of 40 weeks of open‐label treatment, followed by 4‐weeks' off‐treatment follow‐up after being enrolled in PSOARING 1 and 2 in the tapinarof or vehicle arm. Patients could receive tapinarof for up to 52 weeks from PSOARING 1 and 2 start (12 weeks trial) through PSOARING 3 conclusion (40 weeks). In PSOARING 3, patients were treated according to their PGA score. Patients presenting at the beginning of PSOARING 3 with complete disease clearance (PGA = 0 [clear]), discontinued treatment and were monitored for remittive effect (defined as maintenance of a PGA score of 0 [clear] or 1 [almost clear] while off therapy). Patients entering with PGA≥1 maintained application of tapinarof to all affected areas, including newly appearing lesions. All patients that achieved PGA = 0 stopped treatment and were observed for remittive effect off therapy. If a disease flare occurred, defined as PGA ≥ 2, then treatment was re‐started and continued until PGA = 0. At baseline, 10.4% of patients had PGA = 0 [only 2.0% (5/255) were patients previously treated with vehicle]. Overall, 40.9% achieved complete disease clearance (PGA = 0) at least one time during the trial. Also, 58.2% of patients that entered PSOARING 3 with PGA≥2 achieved PGA = 0 or 1 at least once during the trial. No tachyphylaxis was demonstrated for up to 52 weeks. Among patients achieving PGA = 0 at any time during the trial (*n* = 312), the total duration of remittive effect off therapy was a mean of 130.1 days.^32^

### 3.3 Safety

In the phase IIa trial (NCT04042103) (Table 1), the most common adverse effects reported to be related to tapinarof cream were folliculitis (4 patients, 19%) and headache (4 patients, 19%). Other adverse effects were back pain and pruritus. This drug was well tolerated even in sensitive areas. There were no cardiac side effects reported, such as prolongation of QT interval.^27^

In a phase II trial (NCT02564042) (Table 1), the treatment‐emergent adverse effects were more common in the tapinarof groups, but they were tolerable with mild‐to‐moderate severity. The most frequent were folliculitis (9%) and contact dermatitis (3%). Patients in the tapinarof group discontinued the trial due to adverse effects, mainly due to contact dermatitis and application site dermatitis. There were no reports of cardiac or laboratory alterations.^29^

In a phase IIb trial, the adverse effects reported had mild‐to‐moderate severity. It was applied tolerability scores considering the participants and the investigator's point of view for 12 weeks. Participants scored 0 to 1, which means none or slightly site burning and itching on the application site. The investigators scored mostly 0.^30^

In the PSOARING 1 and PSOARING 2 phase III trials (Table 1), adverse effects were more common in the tapinarof group, with 50.3% and 54.5% of the patients, respectively. The most common adverse effects reported were: folliculitis, contact dermatitis, headache and pruritus. In the first trial, one episode of severe folliculitis was reported, and 1.8% of patients in the tapinarof arm discontinued the trial. In the two trials, one severe adverse event of contact dermatitis was reported and led to the discontinuation in 1.5% and 2.0% of patients in trials 1 and 2, respectively. A scale was applied to evaluate burning, stinging, and itching, and it was rated as low in both trials.^31^

Regarding long term data, PSOARING 3 (NCT04053387) showed that after 52 weeks of treatment there were no new safety signals. The most frequent treatment‐emergent adverse events included folliculitis (22.7%), contact dermatitis (5.5%), and upper respiratory tract infection (4.7%). The incidence and severity of folliculitis and contact dermatitis was not worse with long‐term treatment compared with the pivotal trials, and most of these adverse events were mild or moderate.^32^ Trial discontinuation rates due to folliculitis or contact dermatitis were low (1.2% and 1.4%, respectively), and were similar to rates observed in PSOARING I and II.^31^ Tapinarof 1% cream also demonstrated good tolerability, with more than 90% of patients showing no irritation at all visits over the 40‐week period of the trial, and good tolerability on sensitive and intertriginous skin. Also 86%–92% of subjects reported low burning/stinging and itching over 40 weeks. Adherence was high, with the number of doses administered compared with expected exposure being approximately 90%.^32^

## 4 CONCLUSIONS

Psoriasis is a common disease that disrupts daily activities and consequently has a significant impact on quality of life.^33^ Its pathogenesis includes uncontrolled proliferation and abnormal differentiation of keratinocytes, inflammation and vasodilation.^33^

The aryl hydrocarbon receptor is a cytosolic ligand expressed in several skin cell types. AhR signaling is required in healthy skin to maintain skin homeostasis by regulating the skin immunological network, keratinocyte differentiation, skin barrier function, and gene expression in cells. In psoriatic skin, there is a higher expression of AhR.^12^

Tapinarof, an AhR agonist, is a recently FDA approved non‐steroidal topical treatment for plaque psoriasis. Its mechanism of action includes suppression of inflammatory cytokines (in particular IL‐17), the modulation of skin barrier protein expression, and antioxidant activity.^12^

Tapinarof showed to be effective in phase II and phase III randomized controlled trials in patients with mild‐to‐moderate plaque psoriasis. In the phase III trials PSOARING 1 and 2 a PGA response occurred in 35%–40% of patients in the tapinarof group; while only in about 6% of patients in the vehicle group.^31^ These results were sustained in an open label, 40‐week extension trial. In the PSOARING 3 trial, 40.9% of patients achieved complete disease clearance (PGA = 0) and 58.2% of the patients that entered with PGA ≥ 2 achieved PGA = 0 or 1.^32^ This effect was durable, with a mean duration of remittive effect off‐therapy for patients achieving PGA = 0 of 130.1 days. The mechanism for this remittive effect with tapinarof therapy may be explained by its effect through AhR activation which has been shown to (1) inhibit T‐cell expansion and T helper 17 (Th17) cell differentiation and reducing IL‐17 production; (2) promote epigenetic modification of both the forkhead box P3 (FoxP3) and IL‐17 gene promoters, leading to preferential differentiation of regulatory T cells and inhibition of Th17 cells; (3) reduce the in activity of tissue‐resident memory T. This trial also demonstrated good tolerability and high adherence to treatment.^32^

Most adverse events associated with tapinarof were cutaneous, including pruritus, folliculitis, and contact dermatitis. Headache was a frequently reported as adverse effect. Nevertheless, the intensity of these adverse events was mild to moderate with spontaneous resolution and rarely led to the discontinuation of the treatment. No systemic drug accumulation has been reported. There was no increased risk of severe adverse effects, such as cardiac issues or prolongation of QT interval, up to the 52‐week period.^31^, ^32^

Finally, it is important to highlight that treatment adherence to topical therapies is recognized as a critical issue in the management of chronic diseases such as psoriasis, particularly influencing treatment efficacy.^34^ Tapinarof new mechanism of action, will not overcome the adherence problem related to topical administration. Nevertheless, being an effective and safe treatment, its cream formulation and vehicle and simple once a day administration may positively influence adherence to treatment. Naturally, therapeutic education, clear instructions on the use of topical agents, and patient‐supporting interventions are still necessary to improve adherence.^35^

In summary, tapinarof 1% cream has been shown to be effective and safe in the treatment of psoriatic patients, representing an alternative to the current therapeutic options, increasing our armamentarium in the topical treatment of psoriasis.

## AUTHOR CONTRIBUTIONS

Sofia Nogueira, Maria Alexandra Rodrigues, Ron Vender and Tiago Torres had the idea for the article, performed the literature search and data analysis, and drafted and critically revised the work.

## CONFLICT OF INTEREST

Sofia Jacques de Nogueira and Maria Alexandra Rodrigues declare no potential conflict of interest. Ron Vender has been a consultant, and/or scientific advisor, and/or investigator, and/or speaker for Amgen, AbbVie, Arcutis, Astellas, Bausch Health/Valeant, BMS, Boehringer Ingelheim, Celgene, Dermira, Eli Lilly, Galderma, GSK, Janssen, Leo Pharma, Merck (MSD), Merck Serono, Novartis, Pfizer, Regeneron, Roche, Sanofi‐Aventis/Genzyme, Sun Pharma, Takeda and UCB. Tiago Torres declares research grants and/or consulting fees from AbbVie, Almirall, Amgen, Arena Pharmaceuticals, Biocad, Boehringer Ingelheim, Bristol‐Myers Squibb, Celgene, Eli Lilly, Janssen, LEO Pharma, MSD, Novartis, Pfizer, Samsung‐Bioepis, Sandoz, Sanofi, and Viatris.

## Data Availability

The data that support the findings of this study are available from the corresponding author upon reasonable request.
